# Community engagement on-site rapid test for chlamydia and gonorrhea among men who have sex with men: a pioneering study in Guangzhou, China

**DOI:** 10.1186/s12889-022-13460-x

**Published:** 2022-05-24

**Authors:** Xiao-Xin Lin, Si-Yan Meng, Wu-Jian Ke, Xiao-Hui Zhang, Liu-Yuan Wang, Yu-Ying Liao, Han Liu, Pei-Zhen Zhao, Chun-Mei Liang, Hui-Ru Chen, Hai-Ying Long, Bin Yang, Li-Gang Yang

**Affiliations:** 1 Department of Sexually Transmitted Diseases, Dermatology Hospital, Southern Medical University, Guangzhou, 510091 China; 2 UNC Project China, University of North Carolina at Chapel Hill, Guangzhou, China

**Keywords:** Men who have sex with men (MSM), Chlamydia, Gonorrhea, Rapid nucleic acid testing, Community Engagement, Community-based Organization (CBO)

## Abstract

**Background:**

*Chlamydia trachomatis* (CT) and *Neisseria gonorrhoeae* (NG) infections are prevalent among men who have sex with men (MSM) in China. However, compared to syphilis and HIV, the testing rate for chlamydia and gonorrhea remains low. The purpose of this pilot study was to evaluate the feasibility for conducting rapid nucleic acid test for chlamydia and gonorrhea in MSM community-based organizations (CBO).

**Method:**

We recruited our participants through an MSM CBO where free HV and syphilis testing were routinely provided. We collected data including social-demographic background, sexual history, chlamydia and gonorrhea testing history, and reasons for accepting this on-site rapid testing. Urine and/or anorectal swab samples were collected and tested for chlamydia and gonorrhea on-site and the testing results were delivered in about 1.5 h. Positive cases received on-site free treatment.

**Results:**

From August 2020 to October 2020, 634 MSM visited the CBO for syphilis and HIV testing and 158 (158/634, 24.9%) accepted the on-site chlamydia and gonorrhea rapid test, 135 were finally enrolled. The positive rate fo chlamydia was 16.3% (22/135) and 3.0% (4/135) for gonorrhea, respectively. Only 19.3% participants had previously undergone chlamydia and gonorrhea testing and 68.9% (93/135) participants reported that they had heard of gonorrhea, 47.4% (64/135) had heard of chlamydia. The main reason for testing was “free for charge” (66.2%), followed by “convenient, ‘shorter waiting time” (45.2%) and “had high-risk sexual behavior recently” (16.3%).

**Conclusions:**

This pilot study showed that the chlamydia and gonorrhea infection rate remains high among MSM, while the testing rate was low. On-site rapid testing is feasible and potentially preferred by MSM.

## Background

Chlamydia and gonorrhea (CT/NG) are prevalent among men who have sex with men (MSM) with the rectum most likely to be infected, and asymptomatic infections are common [1–3].

A number of studies reported that the prevalence of chlamydia infection was 13.9–22.5% and gonorrhea infection was 5.3–11.0% among Chinese MSM [4, 5]. In order to prevent and control the spread of chlamydia and gonorrhoeae in MSM population, WHO recommended periodic screening in MSM population, and this screening strategy was also recommended by the US CDC and other countries [6, 7].

Despite the high prevalence of chlamydia and gonorrhea infection, chlamydia and gonorrhea testing among MSM is not routinely performed in China. In China, testing for sexually transmitted infections including chlamydia and gonorrhea is dominated by laboratory tests in sexual health clinics, while there are some common problems with getting tested at a sexual health clinic:low awareness to actively conduct chlamydia and gonorrhea testing from either MSM or healthcare providers [8], stigma of visiting STI clinics [9], discomfort with taking or providing a detailed sexual risk assessment and sample collecting, and excessive waiting times and lack of walk-in services [10, 11].These problems are particularly prominent in MSM population for those who experience barriers to receive care at clinic-based facilities [9, 12, 13] .In addition, there is no national guideline to guide the chalmydia and gonorrean screening among MSM population.

Studies suggested that NG/CT point-of-care test is welcomed and community engagement might be useful for promoting HIV and syphilis testing [14, 15]. For Chinese MSM, there are MSM-focused community-based sites serve as an out-of-clinic source of testing, while most of these testing sites only draw blood to test for HIV and syphilis, and do not test for chlamydia and gonorrhea as for lacking of the ability to collect rectal samples. Advances in testing techniques have made self-sampling feasible, reliable and more popular among MSM people [11, 16]. In this study, we worked with a local MSM community-based organization (CBO) to deliver on-site point-of-care chlamydia and gonorrhea testing and treatment in an MSM-friendly environment. The purpose of this preliminary study was to assess the operability of on-site chlamydia and gonorrhea testing and treatment in the MSM community in China.

## Method

### Setting and population

The study was undertaken in a local MSM CBO in Guangzhou, China, named “Lingnan Partner Community Support Center”. This site was chosen because it was developed with strong input from MSM and provided free HIV/syphilis testing to local MSM. The CBO advertised the free chlamydia and gonorrhea testing news to their target MSM who came to the CBO for free HIV/syphilis testing. Eligible MSM made testing appointment through the CBO Internet platform. The eligibility criteria included: male in gender; over 18 years old; self-reported history of anal sex (receptive or penetrative) with another man; no antibiotics use in the past 30 days.

At the MSM CBO site, after receiving their written consent, the eligible MSM were asked to finish an electronic questionnaire survey which included socio-demographic information (age, education, incomes and sexual orientation); sexual behavior information (the number of steady/casual sexual partners in past 6 months, receptive or penetrative and the frequency of condom use); testing history of HIV, syphilis and chlamydia and gonorrhea. We also collected data about reasons for taking the test, the questions we asked including have you ever heard of chlamydia and gonorrhea and the reasons for joining this on-site testing.

### Sample collection and testing

Prior to the start of the project, a GeneXpert automatic medical Polymerase Chain Reaction (PCR) analysis system (GeneXpert IV, Cepheid, Sunnyvale, California, USA) was installed in the MSM CBO. Nucleic acid amplification testing (NAAT) is the preferred method to detect *Chlamydia trachomatis* and *Neisseria gonorrhoeae* [17]. In 2012, the Cepheid Xpert chlamydia and gonorrhea assay, a real-time PCR assay, was approved by FDA to test chlamydia and gonorrhea from female endocervical swabs, vaginal swabs and female and male urine samples [18]. In 2019, the Xpert CT/NG (Cepheid) was further approved by FDA for extragenital diagnostic testing for these infections via throat and rectum samples. Training was provided to the medical personnel and research assistants who involved in the project. After the study began, the engineer from the company regularly carried out quality control and maintenance on the testing instruments.

After completing the survey, the participants were then instructed by a trained staff to collect urine sample or/and rectal swabs by themselves determined by their sexual history. We did not collect pharyngeal samples for corresponding testing because our previous study showed that the positive rate of chlamydia and gonorrhoeae for pharyngeal samples was much lower than that of rectal samples and limitations on the supply of testing reagents [3]. The staff put the samples into a GeneXpert collection kit and turned over 3–4 times to ensure that samples and reagents were well mixed, and then put into machine for testing. Testing results were told by CBO staff or as a message sent to participants in about 90 minutes. Any participants with a positive result would be consulted by a medical staff from STD department of Dermatology Hospital, Southern Medical University who regularly made a visit to the CBO, and free treatment would be provided following the Chinese STI clinical management guidelines. All those who provided samples were informed of the results within two hours, and those who returned on the same day received treatment drugs on the same day. All positive patients were treated in the CBO.

### Data analysis

To establish a database, one investigator entered the questionnaire data and laboratory results into Microsoft Office Excel forms. The IBM SPSS Statistics for Windows Version 23.0 (IBM Corp., Armonk, NY) was used to statistically analyze the dataset. Descriptive statistics were used to present questionnaire survey results and chlamydia and gonorrhea testing results.

### Ethics

This research is part of a clinical trial. Ethical approval for this trial was obtained from the institutional review board at the Southern Medical University Dermatology Hospital (GDDHLS-20190503). All participants signed paper-based consent form before joining the study.

## Results

From August 2020 to October 2020, 158 among 634 MSM who came to the CBO for HIV/syphilis testing accepted the testing invitations. We further excluded 23 MSM on site who did not fully meet the inclusion criteria. Finally 135 were enrolled in the study. Of the 135 participants, the median age was 30 years old (30.43 ± 6.53), and most of them lived in Guangzhou (120/135, 88.9%) with bachelor and above degree (95/135, 70.4%) (Table 1).

**Table 1:** Demographic characteristics and sexual history of 135 study participants attending the community based CT/NG testing in Guangzhou

Characteristic	Number	*%*
**Demographic characteristics**		
Age (years)		
18–30	78	57.8%
> 30	57	42.2%
Local living time		
Less than 6 months	12	8.9%
More than 6 months	123	91.1%
Education		
Middle school or less	3	2.2%
Senior school	6	4.4%
Secondary school or junior collage	31	23.0%
Bachelor or above	95	70.4%
**Sexual partners in past 6 months**		
Number of regular sexual partners		
0 ~ 3	122	90.4%
> 3	13	9.6%
Number of casual sexual partners		
0 ~ 3	101	74.8%
> 3	34	25.2%
**The most recent sex**		
< 30 days	98	72.6%
30 ~ 60 days	25	18.5%
60 ~ 180 days	12	8.9%
Sexual partner		
regular partner	88	65.2%
casual partner	47	34.8%
Anal sex or not		
No	15	11.1%
Yes	120	88.9%
Active inserter	52	43.3%
Passive receiver	36	30%
Active and passive	32	26.7%
Condom use		
Always	88	73.3%
Not all the time	32	26.7%

Of the 135 participants, determined by sexual history, 38 participants only provided rectal samples, 54 only provided urine samples, and 43 provided both rectal and urine samples. Three urine samples were excluded due to incorrect or invalid results from GeneXpert system. Therefore 81 rectal samples and 97 urine samples were finally analyzed.

In total, 22 participants tested positive for chlamydia and 4 participants tested positive for gonorrhea with at least one sample, the overall positive rate was 16.3% (22/135) for chlamydia and 3.0% (4/135) for gonorrhea, respectively (table 2). For rectal samples, the chlamydia positive rate was 22.2% (18/81), and was 4.9% (4/81) for gonorrhea. For urine samples, the chlamydia positive rate was 6.2% (6/97)and was 1.0% (1/97) for gonorrhea. Of the 43 who provided both rectal and urine samples, 1 tested positive for chlamydia in urine sample only, 9 tested positive for chlamydia in rectal samples, only 2 tested positive for chlamydia in both rectal and urine samples, only 1 sample tested positive for gonorrhea in both rectal and urine samples (Table 2).

**Table 2:** The positive rate of chlamydia and gonorrhea among 135 participants by sample type

Sample type	Number of samples	CT + ve (%)	NG + ve (%)	CT + NG + ve (%)
Rectal	81	22.2% (18/81)	4.9% (4/81)	3.70% (3/81)
Urine	97	6.2% (6/97)	1.0% (1/97)	1.0% (1/97)

*CT* chlamydia, *NG* gonorrhea

For the chlamydia and gonorrhea knowledge and testing history,68.9% (93/135) participants reported that they had heard of gonorrhea, 47.4% (64/135) had heard of chlamydia. Only 19.3% participants had previously undergone chlamydia and gonorrhea testing. Regarding on-site testing, in the multiple-choice questions “why are you willing to take this test”, the main reason for accepting the testing invitation was “free for charge” (66.2%), following by “convenient, shorter waiting time,” (45.2%) and “had high-risk sexual behavior recently” (16.3%) (Table 3).

**Table 3:** Chlamydia and gonorrhea knowledge, testing history and reasons for attending the community-based chlamydia and gonorrhea testing (*n* = 135)

Characteristic	Number	*%*
**Knowledge of CT and NG**		
Previously heard of NG	93	68.9%
Previously heard of CT	64	47.4%
**Testing history**		
Previously tested of NG/CT	34	25.2%
**Reasons for attending this testing**		
Free of charge	84	62.2%
Convenient and short waiting time	61	45.2%
Recent risk sex	22	16.3%
Medical staff recommendation	14	10.4%
Friends recommendation	10	7.4%

## Discussion

As far as we know, this study was the first on-site rapid testing for chlamydia and gonorrhea at MSM community in China. The whole process for sample collection, tests, and treatment were all conducted at MSM CBO. Compared with our previous study, 16.3% chlamydia positive rate was in accordance with our previous results, while the 3.0% gonorrhea positive rate was lower than the previous one. This is mainly because the previous study recruited participants from both STI clinic and MSM community [3]. The results further proved that chlamydia and gonorrhea were prevalent among MSM in Guangzhou. This study also found that despite the high prevalence of chlamydia and gonorrhea infection, more than half of participants had never heard of chlamydia, and only about 1/4 of them had been tested. We also investigated the reasons for taking the tests, beside the free of charge, almost half of participants thought CBO-based on-site testing was convenient and acceptable. This result informs us that on-site CBO-based testing is feasible.

WHO recommends asymptomatic MSM need to be tested for urethral and anorectal chlamydia and gonorrhea [6]. Our previous study along with this one support that regular screening of chlamydia and gonorrhea is necessary for the asymptomatic MSM in Guangzhou. Unfortunately, only minority of MSM had ever been tested in Guangzhou. Lack of awareness among MSM and healthcare providers, limited testing resources and facility capacity are all barriers to expand the tests. In addition, MSM may also be reluctant to go to medical facilities for testing because of possible discrimination [19]. In this pilot study, all the testing process were conducted at CBO, with the minimal training operator, occupied very small space, and provided results in a much shorter time (90 mins) than traditional test [18]. In addition, we provided treatment for positive participants on site, which is important to prevent the further transmission. Nearly half of the participants admitted that they came for the test as it was convenient and had shorter waiting time, proving that such a model of testing was attractive to the MSM.

This pilot study proved that on-site testing was feasible, but since there was no control group, it could not be concluded whether on-site testing was more popular with MSM although our questionnaire results showed that half of the participants consider on-site testing acceptable. One of the other limitations of this study is that we did not calculate the sample size in advance. Although the positive rate obtained was similar to that of our previous studies, the potential bias could not be ruled out.

## Conclusion

Our preliminary findings demonstrated that chlamydia and gonorrhea tests at MSM CBO is feasible, while as we did not conduct a cost–benefit analysis, the sustainability of such testing is unclear. Multi-site studies, including cost–benefit evaluations, are needed before such model of testing could be implemented widely in Guangzhou or even throughout China.

## Data Availability

The datasets generated during and analyzed during the current study are not publicly available as following the hospital procedure but are available from the corresponding author on reasonable request.

## Abbreviations

CT: Chlamydia Trachomatis
NG: Neisseria Gonorrhoeae
MSM: Men who have Sex with Men
CBO: Community-based organization
HIV: Human Immunodeficiency Virus
PCR: Polymerase Chain Reaction
STD: Sexually Transmitted Diseases
WHO: World Health Organization
